# FGF/FGFR1 system in paired breast tumor-adjacent and tumor tissues, associations with mammographic breast density and tumor characteristics

**DOI:** 10.3389/fonc.2023.1230821

**Published:** 2023-07-20

**Authors:** Öykü Boraka, Marie Klintman, Johan Vallon-Christersson, Sophia Zackrisson, Per Hall, Signe Borgquist, Ann H. Rosendahl

**Affiliations:** ^1^ Department of Clinical Sciences Lund, Oncology, Lund University, Skåne University Hospital, Lund, Sweden; ^2^ Department of Clinical Sciences Lund, Oncology, Lund University, Lund, Sweden; ^3^ Department of Translational Medicine, Diagnostic Radiology, Lund University, Malmö, Sweden; ^4^ Department of Medical Epidemiology and Biostatics, Karolinska Institutet, Stockholm, Sweden; ^5^ Department of Oncology, Södersjukhuset, Stockholm, Sweden; ^6^ Department of Oncology, Aarhus University, Aarhus University Hospital, Aarhus, Denmark

**Keywords:** breast cancer, mammographic breast density, fibroblast growth factor receptor, FGFR1, tumor characteristics

## Abstract

**Introduction:**

Mammographic breast density (MBD) is an established breast cancer risk factor, yet the underlying molecular mechanisms remain to be deciphered. Fibroblast growth factor receptor 1 (FGFR1) amplification is associated with breast cancer development and aberrant FGF signaling found in the biological processes related to both high mammographic density and breast cancer microenvironment. The aim of this study was to investigate the FGF/FGFR1 expression in-between paired tumor-adjacent and tumor tissues from the same patient, and its associations with MBD and tumor characteristics.

**Methods:**

FGFR1 expression in paired tissues from 426 breast cancer patients participating in the Karolinska Mammography Project for Risk Prediction of Breast Cancer (KARMA) cohort study was analyzed by immunohistochemistry. FGF ligand expression was obtained from RNA-sequencing data for 327 of the included patients.

**Results:**

FGFR1 levels were differently expressed in tumor-adjacent and tumor tissues, with increased FGFR1 levels detected in 58% of the tumors. High FGFR1 expression in tumor tissues was associated with less favorable tumor characteristics; high histological grade (OR=1.86, 95% CI 1.00–3.44), high Ki67 proliferative index (OR=2.18, 95% CI 1.18–4.02) as well as tumors of Luminal B-like subtype (OR=2.56, 95%CI 1.29–5.06). While no clear association between FGFR1 expression and MBD was found, FGF ligand (FGF1, FGF11, FGF18) expression was positively correlated with MBD.

**Discussion:**

Taken together, these findings support a role of the FGF/FGFR1 system in early breast cancer which warrants further investigation in the MBD–breast cancer context.

## 1 Introduction

Breast cancer is the most frequently diagnosed cancer in women worldwide with a steady increase in global incidence (1). While the heterogeneity of the disease constitutes a major challenge in breast cancer treatment (2), breast cancer prevention has gained increasing attention as up to 30% of all breast cancer cases are estimated to be preventable through combined modification of breast cancer risk factors including low physical activity, high body mass index (BMI), alcohol consumption, and smoking (3, 4).

Mammographic breast density (MBD), is a well-established breast cancer risk factor that is assessed *via* mammography images, and percent density is defined as the relative abundance of fibroglandular tissue (dense area) relative to fat (adipose) tissue (non–dense area) (5). In women with the highest (≥75%) MBD, a nearly 5-fold increased risk of breast cancer was seen when compared with women with the lowest (<10%) MBD (6). MBD is proposed to be a modifiable breast cancer risk factor, rendering MBD reduction a potential preventive measure against breast cancer (7–9). Although MBD is associated with breast cancer risk, it is unclear how. Changes in the local microenvironment of the breast, within the fibroglandular [e.g., paracrine signaling and extracellular matrix (ECM) remodeling (10)] and adipose (e.g., adipocyte secretome (11)) tissue compartments, are known to impact breast cancer initiation and progression. High abundance of an active stromal compartment thus provides a tumor promoting niche where the extracellular matrix further acts as a structural grit for tumor formation and a reservoir for growth factors, such as fibroblast growth factors (FGFs), to stimulate tumor-initiating epithelial cells (12).

FGF signaling is shared between the biological processes relating to both high MBD and breast cancer microenvironment (12, 13). FGF receptors (FGFRs) are members of the receptor tyrosine kinase family of transmembrane receptors and consist of four members that are encoded by four distinct genes: FGFR1–4. Upon activation by fibroblast growth factor ligands (FGF1–23), FGFRs phosphorylate their intracellular substrates (e.g., FGFR substrate 2 (FRS2), phospholipase Cγ (PCγ)) (14) and trigger signal transduction pathways that have key roles in mediating cell proliferation, differentiation, migration, and apoptosis as well as in the pathophysiology of a wide spectrum of diseases including cancers (15).

*FGFR* aberrations, including amplifications, mutations, and rearrangements, exist in various types of human cancers at an average frequency of ~7% (16). Relative to other cancer types, *FGFR* aberrations are more frequent in breast cancer (18%), with *FGFR1* amplification being the predominant aberration observed in 14% of breast cancer patients (16). The *FGFR1* is an amplified oncogene associated with the early evolution of breast cancer clones (17). The importance of the FGF/FGFR system in breast cancer is additionally reinforced *via* genome-wide association studies where common genetic variation in the *FGFR2* locus is robustly associated with breast cancer (18). *FGFR1* amplification is further found associated with antiestrogen resistance in estrogen receptor positive (ER+) tumors (19). An *in vitro* study that used a fibroblast-derived ECM scaffold showed that FGF2, a FGFR1 ligand, mediates estrogen-independent ER signaling in breast cancer cells when bound to the ECM, rendering FGFR1 as a relevant MBD–related target (12).

Despite the clinical evidence of an association between MBD and breast cancer risk, the molecular mediators or involved pathways are not known. In this study, we investigated FGFR1 gene and protein and *FGF* ligand expression in paired tumor-adjacent and tumor tissues in relation to MBD and tumor characteristics by making use of a population-based prospective screening cohort, The Karolinska Mammography Project for Risk Prediction of Breast Cancer (KARMA) cohort, with the aim of contributing to bridge the knowledge gaps in the MBD–breast cancer association.

## 2 Methods

### 2.1 Study population

**Figure 1:**
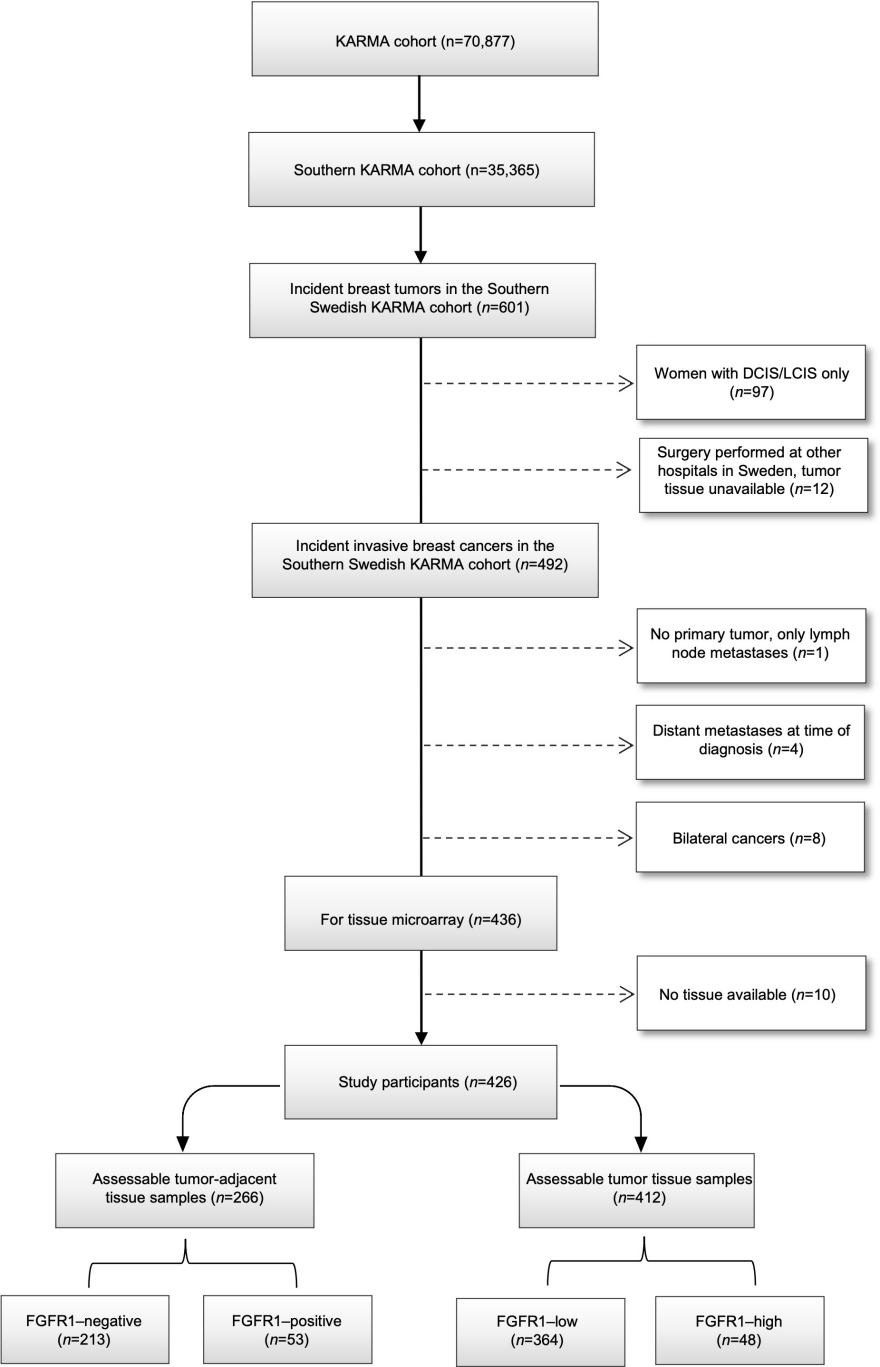
Consort flow diagram of included and excluded individuals within final study population.

The Karolinska Mammography Project for Risk Prediction of Breast Cancer (KARMA) is a prospective cohort study with 70,877 participants who attended mammography screening (aged 40–74 years) or clinical mammography at four hospitals in Sweden in 2011–2013, 35,367 of whom were enrolled in Southern Sweden (20). Baseline characteristics were collected *via* self-reported questionnaires. Among the participants in the Southern Swedish KARMA cohort, 601 incident breast cancers occurred by December 31, 2016. For tissue microarray (TMA) construction, patients with ductal/lobular carcinoma *in situ* (n=97), patients who underwent surgery at other hospitals in Sweden (n=12), patients with occult breast cancer, i.e., undetectable primary tumor only lymph node metastasis (n=1), patients with distant metastases at primary diagnosis (n=4), or bilateral cancers (n=8) were excluded. Paired tumor-adjacent (normal) and tumor tissues from the remaining 426 patients with invasive breast cancer and available tissue were collected and compiled in TMAs for subsequent protein expression analysis (**Figure 1**). The study was approved by the regional Ethics Committee at Karolinska Institutet, Sweden (Dnr 2010/958-31/1 and 2013/2090-32), and all study participants signed informed consent.

### 2.2 Patient and tumor characteristics

Patient and tumor characteristics at time of diagnosis were obtained from the Swedish National Quality Register for Breast Cancer (NKBC) and validated through assessment of medical records (MK). Pathology data included tumor size (≤20 mm versus >20 mm), lymph node status (negative/positive), distant metastasis (no/yes), Nottingham histological grade (1 + 2 versus 3), estrogen receptor (ER)-, progesterone receptor (PR)-, human epidermal growth factor receptor 2 (HER2)-status (positive/negative), and Ki67 (% positive cells, low + intermediate versus high, according to cut-off definitions below). Breast cancer subtypes were defined according to the Swedish regional guidelines for clinical decision making over three time periods. In 2011–2013, Luminal A-like subtype was defined as ER >10% tumors with i) histological grade 1, or ii) histological grade 2 and Ki67 ≤20%. Luminal B-like subtype was defined as ER >10% tumors with i) histological grade 3, or ii) histological grade 2 and Ki67 >20% in all four participating pathology laboratories. The 20% Ki67 cut-off was defined to identify the 7th decile, associated with prognosis in patients with lymph node negative, histological grade 2, ER+, HER2- tumors, as previously described (21). In 2014–2015, the cut-off to define high versus low Ki67 was internally re-validated to identify the 7th decile, resulting in different cut-offs for high Ki67 in the four laboratories: Helsingborg (>30%) and Lund, Malmö and Kristianstad (>20%). Luminal A-like was then defined as ER >10%, PR >20% tumors with i) histological grade 1, or ii) histological grade 2 and low Ki67. Luminal B-like was defined as ER >10% and i) histological grade 3, or ii) histological grade 2 and high Ki67 or PR ≤20%. From 2015 and onwards, Luminal A-like was defined as ER >10% tumors with i) histological grade 1, or ii) histological grade 2 and Ki67 <20%, or iii) histological grade 2, Ki67 20–30% and PR >20%. Luminal B-like subtype was defined as ER >10% tumors with i) histological grade 3, or ii) histological grade 2 and Ki67 >30%, or iii) histological grade 2, Ki67 20–30% and PR ≤20%. For all time periods, the HER2+ subtype was defined as HER2+ tumors regardless of other characteristics, and triple-negative breast cancer (TNBC) as ER-negative (≤ 10%), PR-negative (≤ 10%) and HER2-negative.

### 2.3 Mammographic breast density measurement

Raw digital full field mammograms from mediolateral oblique views of both breasts were collected at KARMA study recruitment for MBD assessment. Absolute dense area (cm^2^) was measured on raw mammograms by using the density measurement tool iCAD (iReveal^®^, Nashua, NH, USA) and fully automated STRATUS method (22). Percent dense area was calculated by dividing absolute dense area by total breast area and categorized based on Breast Imaging Reporting and Data System 5^th^ edition (BI-RADS; American College of Radiology, Reston, VA, USA): category A (almost entirely fatty breasts), category B (scattered areas of fibroglandular density), category C (heterogeneously dense breasts), category D (extremely dense breasts) (23).

### 2.4 Tissue microarray construction and immunohistochemistry

**Figure 2:**
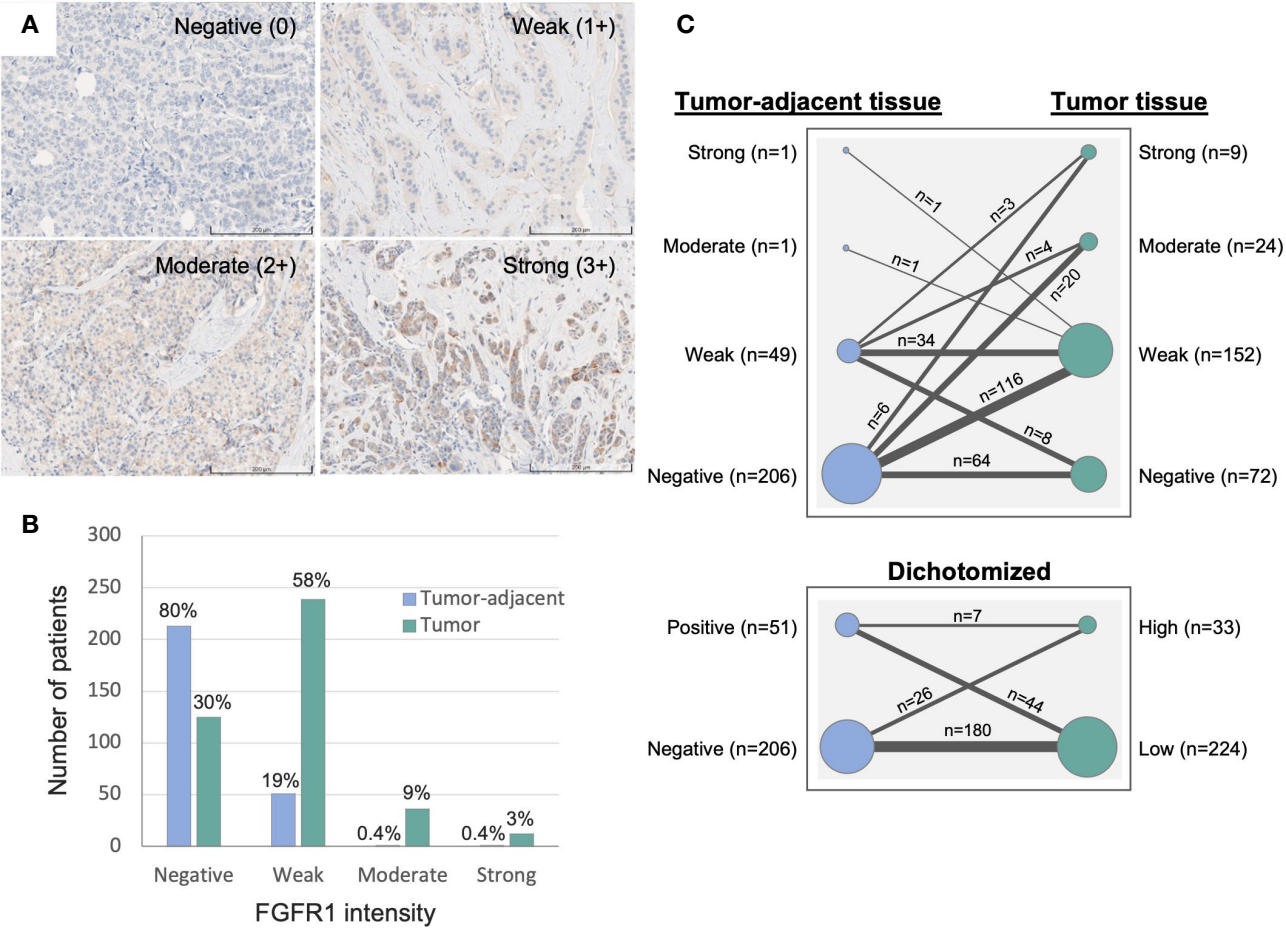
Immunohistochemistry evaluation of FGFR1 expression in tumor-adjacent and tumor tissues. **(A)** Representative images of immunohistochemically stained FGFR1 in tumor tissue and cytoplasmic intensity levels: Negative (0), Weak (1+), Moderate (2+), Strong (3+) expression. **(B)** Graph displaying the distribution of FGFR1 expression in tumor-adjacent (n=266) and tumor tissues (n=412). **(C)** Graph displaying the change in FGFR1 expression between paired tumor-adjacent and tumor tissues (n=257), according to four levels of FGFR expression (top panel) or dichotomized FGFR1 expression levels (bottom panel).

Duplicate 1 mm tissue cores were collected from formalin-fixed and paraffin-embedded tissue blocks and mounted in recipient paraffin blocks for tissue microarray (TMA) construction. TMAs with paired tissues were then cut into 4 µm sections, de-paraffinized and pre-treated for antigen retrieval with Cell Conditioning 1 (CC1) buffer at pH 7.8 (#950-500, Roche). Immunohistochemistry staining was performed using monoclonal anti-FGFR1 antibody (#9740, Cell Signaling Technology, validation performed on kidney/tonsil/breast cancer tissues *via* IHC) and visualized by ChromoMap DAB Kit (#760-159, Roche) with the use of the fully automated DISCOVERY ULTRA IHC research platform (Ventana). The stained TMAs were then scanned for image digitalization and analyzed for their FGFR1 expression by using PathXL Xplore software (version 5.1.1, Philips). Cytoplasmic intensity of FGFR1 staining in breast epithelial and cancer cells were scored from 0–3, representing negative (0), weak (1+), moderate (2+), strong expression (3+), respectively (**Figure 2**). The FGFR1 staining evaluation was performed in two independent readings (ÖB), blinded to patient and tumor characteristics. For discordant cases, another round of reading was performed, and final values were decided. Reporting Recommendations for Tumor Marker Prognostic Studies (REMARK) were followed throughout the study (24).

### 2.5 Gene expression data

Gene expression data from 327 (77%) of 426 tumors were available and retrieved from the Sweden Cancerome Analysis Network Breast (SCAN-B) Initiative (25). Briefly, gene expression profiles of individual samples were analyzed *via* whole transcriptome mRNA–sequencing (>19,000 genes) with the use of Illumina sequencers, and subsequent pre–processing and log_2_ transformation of the RNA-seq data. Robust single sample predictor (SSP) models were trained for four RNA-sequencing-based molecular subtypes of breast cancer: Luminal A, Luminal B, HER2-enriched, and Basal-like, as described elsewhere (26).

### 2.6 Statistical analyses

Distribution of FGFR1 protein expression levels were analyzed in relation to patient and tumor characteristics. FGFR1 expression was dichotomized based on staining intensities into FGFR1-negative (intensity 0) and FGFR1-positive (weak-strong, intensities 1–3) in tumor adjacent tissues; FGFR1-low (negative-weak, intensities 0–1) and FGFR1-high (moderate-strong, intensities 2–3) in tumor tissues. Only two patients displayed moderate-strong FGFR1 levels in tumor adjacent tissue, hence, dichotomization was conducted differently in tumor-adjacent and tumor tissues to enable statistical analyses of comparably sized groups. Cohort-specific relative values from mean-centered, log_2_-transformed *FGFR1* and *FGF* ligand expression data were used as continuous variables. Distributional differences between FGFR1 levels in tumor-adjacent and tumor tissues in relation to patient and tumor characteristics were analyzed by chi-square test. Associations of FGFR1 expression with patient and tumor characteristics were examined by logistic regression providing odds ratios (OR) with 95% confidence intervals (95%CI) in crude and adjusted models (adjustment by age at diagnosis and tissue storage time that can influence protein levels). Due to few patients in the lowest (A) or highest (D) MBD categories, dichotomized BI-RADS categories were used (A+B = low MBD; C+D = high MBD) for odds ratio assessment. Associations between *FGFR1* and *FGF* ligand expression (*FGF1–11, FGF16–23*) with MBD were examined by Joncksheere-Terspstra test. *FGF* ligands that did not have quantifiable gene expression values for the majority of the cohort (*FGF3, 4, 6, 8, 17, 21, 23*) were not included in the final analyses, therefore, not presented. All statistical analyses were conducted using SPSS (versions 27 and 29 for Mac, IBM).

## 3 Results

### 3.1 Patient characteristics and FGFR1 expression in paired tumor-adjacent and tumor tissues

Median time from study inclusion to breast cancer diagnosis was 24.6 months (inter quartile range, IQR; 2.8–38.7). The majority of patients were >50 years at diagnosis, and the median age at diagnosis among patients with tissue in the TMA and with available gene expression data was 62 and 61 years, respectively (**Table 1**). Of the 426 patients included in the study, 412 (97%) had assessable FGFR1 levels in tumor tissue, 266 (62%) in paired tumor-adjacent tissue, and 327 (77%) had available gene expression data. Overall, FGFR1-positivity was detected more frequently and at higher levels in breast cancer tissues compared with paired tumor-adjacent tissues (**Figure 2**). Among the tumor-adjacent tissue samples with assessable FGFR1 levels, 53 (20%) displayed positive staining, of which 2 (1%) expressed moderate-high levels (**Figure 2B**). Of the corresponding tumor tissue samples, 287 (70%) displayed positive FGFR1 staining, of which 48 (12%) expressed moderate-high levels (**Figures 2A, B**). In total, 257 patients had assessable FGFR1 staining in both tumor-adjacent and tumor tissue, of whom 98 (38%) had stable FGFR1 levels that were maintained between tumor-adjacent and tumor tissues, 149 (58%) displayed higher FGFR1 levels in tumor tissue compared with tumor-adjacent tissue, while 10 (4%) had lower FGFR1 levels in tumor relative to tumor-adjacent tissue (**Figure 2C**).

**Table 1:** Distribution of patient characteristics among included breast cancer patients with protein (TMA-IHC) or gene expression (GEX) data.

	All women	
	TMA-IHC	GEX
	(n=426)	(n=327)
Age at baseline, median (IQR)	60 (51–67)	60 (51–67)
Age at diagnosis, median (IQR)	62 (52-68)	61 (52–68)
Age at diagnosis, years (%)		
≤50 years (premenopausal)	78 (18)	62 (19)
>50 years (postmenopausal)	348 (82)	265 (81)
ANTHROPOMETRY		
BMI at baseline, kg/m^2^, median (IQR)	25 (23–28)	25 (23–28)
BMI at diagnosis, kg/m^2^, median (IQR)	25 (23–28)	25 (23–28)
BMI at diagnosis (%)		
<25.0	220 (52)	169 (52)
≥25.0	206 (48)	158 (48)
BI-RADS category for breast density (%)		
A, almost entirely fatty	24 (6)	16 (5.0)
B, scattered areas of fibroglandular density	154 (36)	113 (35.0)
C, heterogeneously dense	199 (47)	163 (50.5)
D, extremely dense	44 (10)	31 (9.6)
LIFESTYLE AND REPRODUCTIVE FACTORS		
Age at menarche, median (IQR)	13 (12–14)	13 (12–14)
Missing (%)	32 (8)	24 (7)
No. of births (%)		
0	41 (10)	32 (10)
1	63 (15)	48 (15)
2	209 (49)	169 (52)
3	78 (18)	53 (16)
≥4	13 (5)	9 (3)
Age at first child birth (%)		
≤20	45 (11)	37 (11)
21-25	118 (28)	83 (25)
26-30	125 (29)	96 (29)
>30	75 (18)	63 (19)
Nulliparous	41 (10)	32 (10)
Missing	22 (5)	16 (5)
Oral contraceptive use (%)		
No	82 (19)	66 (20)
Yes	319 (75)	243 (74)
issing	25 (6)	18 (6)
Hormone replacement therapy use (%)		
No	243 (57)	191 (58)
Yes	154 (36)	114 (35)
Missing	29 (7)	22 (7)
Alcohol consumption (%)		
No	62 (15)	54 (17)
Yes	339 (80)	254 (78)
Missing	25 (5)	19 (5)
Alcohol, gram per week, median (IQR)	37 (12–63)	37 (12–63)

Number and valid column % presented unless specified otherwise. Missing reported if >1%, shown as total percentages.

TMA-IHC, Tissue microarray-immunohistochemistry; GEX, Gene expression.

### 3.2 Distribution of FGFR1 levels according to patient and tumor characteristics

Frequency distribution of FGFR1 levels in tumor-adjacent and tumor tissue according to patient and tumor characteristics are shown in **Table 2**. Compared to patients with FGFR1 negative tumor-adjacent tissue, patients with FGFR1 positive tumor-adjacent tissue were more frequent >50 years at diagnosis. No difference in the FGFR1 tumor levels was seen in relation to age at diagnosis. A similar distribution of FGFR1 levels in both tumor-adjacent and tumor tissues was seen across BMI categories at diagnosis. Among patients with FGFR1 positive tumor-adjacent tissue, 56% had high MBD (BI-RADS C-D) compared with 63% of patients with FGFR1-negative expression. Conversely, among patients with FGFR1-high tumors, 65% had high MBD (BI-RADS C-D) compared to 57% of patients with FGFR1-low tumors. Overall, there was no distinct difference in the FGFR1 frequencies across tumor characteristics between FGFR1-negative and positive expression groups in tumor-adjacent tissues except for the few patients that with positive FGFR1 levels in tumor-adjacent tissue appeared more likely to have progesterone receptor (PR) positive tumors. Patients with FGFR1-high tumors were more likely to have tumors of histological grade 3 and high Ki67 proliferative index, compared with patients with FGFR1-low tumors. Furthermore, compared with patients with FGFR1-low tumors, patients with FGFR1-high tumors were more likely to have Luminal B-like tumors.

**Table 2:** Distribution of patient and tumor characteristics based on FGFR1 expression in tumor-adjacent and tumor tissues.

	All	Tumor-adjacent tissue (IHC)			Tumor tissue (IHC)		
		Negative	Positive	*p*-value	Low	High	*p-*value
	(n=426)	(n=213)	(n=53)		(n=364)	(n=48)	
PATIENT CHARACTERISTICS							
Age at diagnosis (median, IQR)	62 (52-68)	61 (51–68)	58 (53–66)		62 (53–68)	61 (51–68)	
Age at diagnosis, years (%)							
≤50 years (premenopausal)	78 (18)	52 (24)	6 (11)		66 (18)	10 (21)	
>50 years (postmenopausal)	348 (82)	161 (76)	47 (89)	**0.039**	298 (82)	38 (79)	0.650
BMI at diagnosis (%)							
BMI at diagnosis, kg/m^2^, median (IQR)	25 (23–28)	25 (23–27)	25 (22–29)		25 (23–28)	24 (22–30)	
<25.0	220 (52)	124 (58)	28 (52)		184 (51)	29 (60)	
≥25.0	206 (48)	89 (42)	25 (47)	0.478	180 (49)	19 (40)	0.198
BI-RADS category for breast density (%)							
A, almost entirely fatty	24 (6)	7 (3)	4 (8)		18 (5)	4 (8)	
B, scattered fibroglandular density	154 (36)	69 (32)	19 (36)		137 (38)	13 (27)	
C, heterogeneously dense	199 (47)	113 (52)	16 (30)		165 (46)	28 (58)	
D, extremely dense	44 (10)	23 (11)	14 (26)	**0.003**	39 (11)	3 (6)	0.211
TUMOR CHARACTERISTICS							
Tumor size (%)							
≤20 mm (T1)	332 (78)	167 (79)	44 (83)		284 (78)	37 (79)	
≥21mm (T2+)	93 (22)	45 (21)	9 (17)	0.493	80 (22)	10 (21)	0.913
Nodal status (%)							
Negative (N0)	306 (72)	155 (73)	38 (72)		257 (71)	35 (73)	
Positive (N+)	120 (28)	58 (27)	15 (28)	0.876	107 (29)	13 (27)	0.740
Histological grade (%)							
1 + 2	291 (69)	146 (69)	39 (75)		253 (70)	26 (55)	
3	133 (31)	67 (31)	13 (25)	0.363	110 (30)	21 (45)	**0.047**
Ki67 (%)							
Low + intermediate	253 (60)	124 (59)	37 (70)		221 (61)	20 (42)	
High	172 (40)	88 (41)	16 (30)	0.131	142 (39)	28 (58)	0.011
ER status (%)							
Positive	381 (90)	189 (89)	51 (96)		326 (90)	42 (88)	
Negative	44 (10)	23 (11)	2 (4)	0.115	37 (10)	6 (12)	0.624
PR status (%)							
Positive	314 (74)	159 (75)	47 (89)		272 (75)	32 (67)	
Negative	111 (26)	53 (25)	6 (11)	**0.032**	91 (25)	16 (33)	0.220
HER2 status (%)							
Negative	384 (90)	192 (91)	47 (89)		329 (91)	42 (88)	
Positive	41 (10)	20 (9)	6 (11)	0.680	34 (9)	6 (12)	0.491
SURROGATE SUBTYPES (IHC)							
Luminal A-like	232 (54)	118 (55)	34 (64)		205 (56)	17 (35)	
Luminal B-like	123 (29)	58 (27)	13 (25)		99 (27)	21 (44)	
HER2-positive	41 (10)	20 (9)	6 (11)		34 (9)	6 (12)	
Triple-negative (TNBC)	30 (7)	17 (8)	0 (0)	0.166	26 (7)	4 (8)	**0.047**
MOLECULAR INTRINSIC SUBTYPES (RNAseq)							
Luminal A	201 (61)	102 (61)	30 (81)		181 (63)	17 (49)	
Luminal B	61 (19)	27 (16)	7 (19)		52 (18)	8 (23)	
HER2-enriched	38 (12)	23 (14)	0 (0)		32 (11)	5 (14)	
Basal-like	28 (8)	15 (9)	0 (0)	**0.015**	23 (8)	5 (14)	0.372
Missing	98 (23)	46 (22)	16 (30)		76 (21)	13 (27)	

All data presented as numbers and valid column percentages. Missing reported if >1%, shown as total percentages.

Values in bold indicate p<0.05.

### 3.3 Associations between FGFR1 levels, mammographic breast density and tumor characteristics

The probability of FGFR1-positivity in tumor-adjacent tissues or FGFR1-high levels in tumor tissues was assessed between patients with low and high MBD. No clear association between FGFR1 expression in tumor-adjacent or tumor tissue and MBD was found (**Table 3**). FGFR1-high tumors were associated with histological grade 3 (OR=1.86, 95% CI 1.00–3.44), high Ki67 (OR=2.18, 95% CI 1.18–4.02), and Luminal B-like tumors (OR=2.56, 95% CI 1.29–5.06) (**Table 3**). When adjusted for age at diagnosis and number of years the samples were stored, all associations remained essentially the same.

**Table 3:** Associations between patient and tumor characteristics and FGFR1 expression in tumor-adjacent and tumor tissues.

	All	Tumor-adjacent tissue (IHC)		Tumor tissue (IHC)	
	n=426	OR (95% CI)^a^	OR (95% CI)^b^	OR (95% CI)^a^	OR (95% CI)^b^
PATIENT CHARACTERISTICS					
Age at diagnosis					
≤50 years (premenopausal)	78 (18)	1.00 (REF)	1.00 (REF)	1.00 (REF)	1.00 (REF)
>50 years (postmenopausal)	348 (82)	**2.53 (1.02–6.26)**	**8.00 (2.47–25.86)**	0.84 (0.40–1.78)	1.08 (0.36–3.28)
BMI at time of diagnosis, kg/m^2^					
<25.0	220 (52)	1.00 (REF)	1.00 (REF)	1.00 (REF)	1.00 (REF)
≥25.0	206 (48)	1.24 (0.68–2.28)	1.26 (0.69–2.32)	0.67 (0.36–1.24)	0.66 (0.36–1.23)
BI-RADS category for breast density					
A+B (low density)	178 (42)	1.00 (REF)	1.00 (REF)	1.00 (REF)	1.00 (REF)
C+D (high density)	243 (57)	0.73 (0.40–1.34)	0.68 (0.36–1.30)	1.39 (0.74–2.60)	1.34 (0.70–2.60)
TUMOR CHARACTERISTICS					
Tumor size					
≤20 mm (T1)	332 (78)	1.00 (REF)	1.00 (REF)	1.00 (REF)	1.00 (REF)
≥21mm (T2+)	93 (22)	0.76 (0.35–1.67)	0.78 (0.35–1.72)	0.96 (0.46–2.01)	0.93 (0.44–1.96)
Nodal status					
Negative (N0)	306 (72)	1.00 (REF)	1.00 (REF)	1.00 (REF)	1.00 (REF)
Positive (N1+)	120 (28)	1.06 (0.54–2.06)	1.08 (0.55–2.11)	0.89 (0.45–1.75)	0.86 (0.44–1.70)
Histological grade					
1 + 2	291 (69)	1.00 (REF)	1.00 (REF)	1.00 (REF)	1.00 (REF)
3	133 (31)	0.73 (0.36–1.45)	0.75 (0.37–1.51)	**1.86 (1.00–3.44)**	1.83 (0.98–3.40)
Ki67 status					
Low + intermediate	253 (60)	1.00 (REF)	1.00 (REF)	1.00 (REF)	1.00 (REF)
High	172 (40)	0.61 (0.32–1.16)	0.62 (0.32–1.21)	**2.18 (1.18–4.02)**	**2.09 (1.13–3.88)**
ER status					
Positive	381 (90)	1.00 (REF)	1.00 (REF)	1.00 (REF)	1.00 (REF)
Negative	44 (10)	0.32 (0.07–1.41)	0.33 (0.08–1.46)	1.26 (0.50–3.16)	1.26 (0.50–3.18)
PR status					
Positive	314 (74)	1.00 (REF)	1.00 (REF)	1.00 (REF)	1.00 (REF)
Negative	111 (26)	**0.38 (0.16–0.95)**	**0.39 (0.16–0.98)**	1.50 (0.78–2.85)	1.49 (0.78–2.86)
HER2 status					
Negative	384 (90)	1.00 (REF)	1.00 (REF)	1.00 (REF)	1.00 (REF)
Positive	41 (10)	1.23 (0.47–3.22)	1.26 (0.48–3.35)	1.38 (0.55–3.49)	1.29 (0.51–3.28)
SURROGATE SUBTYPES (IHC)					
Luminal A-like	232 (54)	1.00 (REF)	1.00 (REF)	1.00 (REF)	1.00 (REF)
Luminal B-like	123 (29)	0.78 (0.38–1.59)	0.81 (0.39–1.69)	**2.56 (1.29–5.06)**	**2.49 (1.25–4.98)**
HER2-positive	41 (10)	1.04 (0.39–2.80)	1.07 (0.39–2.93)	2.13 (0.78–5.78)	1.99 (0.73–5.46)
Triple-negative (TNBC)	30 (7)	–	–	1.86 (0.59–5.94)	1.90 (0.59–6.10)
MOLECULAR INTRINSIC SUBTYPES (RNAseq)					
Luminal A	201 (61)	1.00 (REF)	1.00 (REF)	1.00 (REF)	1.00 (REF)
Luminal B	61 (19)	0.88 (0.35–2.22)	0.84 (0.33–2.14)	1.64 (0.67–4.00)	1.75 (0.71–4.33)
HER2-enriched	38 (12)	–	–	1.67 (0.57–4.83)	1.56 (0.53–4.60)
Basal-like	28 (8)	–	–	2.32 (0.78–6.87)	2.35 (0.78–7.04)

a Crude model.

b Model adjusted for age at diagnosis and number of years the samples were stored.

Values in bold indicate p<0.05.

### 3.4 Correlation between FGFR1 gene and protein expression

**Figure 3:**
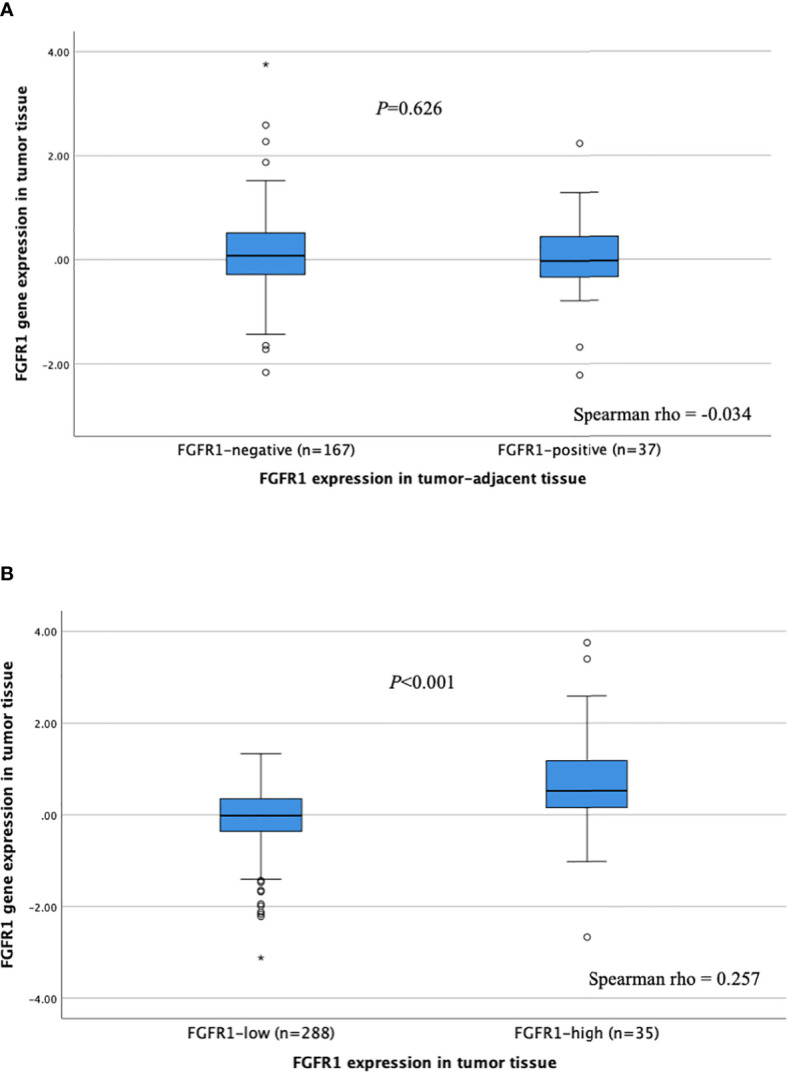
*FGFR1* expression according to FGFR1 levels in **(A)** tumor-adjacent and **(B)** tumor tissues. *P*-values from Mann Whitney U-test comparing relative *FGFR1* expression according to tumor-adjacent FGFR1-negative vs. positive tissue and tumor FGFR1-low vs high tissue, respectively. Spearman rho coefficients shown for correlation assessment between FGFR1 gene and protein expression. Open circles and stars indicate outliers and extremes, respectively.

The correlation between *FGFR1* expression (mRNA) in tumor tissues and FGFR1 expression in corresponding tumor-adjacent and tumor tissues was investigated (**Figure 3**). No correlation between *FGFR1* gene expression and protein levels was observed for tumor-adjacent tissues (Spearman rho -0.034), and the relative *FGFR1* expression was similar among patients that displayed negative versus positive FGFR1 expression (*P*=0.626, **Figure 3A**). In tumor tissues, a weak positive correlation was found between FGFR1 gene and protein expression (Spearman rho 0.257), and the relative *FGFR1* expression was overall higher in FGFR1-high tumors compared with FGFR1-low tumors (*P*<0.001, **Figure 3B**).

### 3.5 Distribution of *FGFR1* and *FGF* ligand expression in relation to mammographic breast density

**Figure 4:**
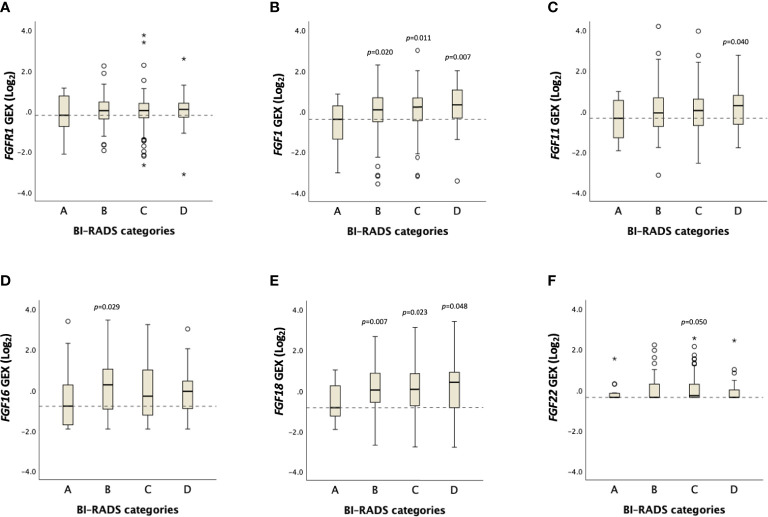
Distribution of *FGFR1* and *FGF* ligand expression across BI-RADS MBD categories. Box plots showing Log_2_ transformed gene expression (GEX) values of **(A)** *FGFR1*, **(B)** *FGF1*, **(C)** *FGF11*, **(D)** *FGF16*, **(E)** *FGF18*, **(F)** *FGF22* in breast tumor tissues across BI-RADS MBD categories. *P*-values comparing differences between groups in relation to BI-RADS A, assessed by the Jonckheere-Terpstra test. Dashed lines represent the median values of BI-RADS A. Open circles and stars indicate outliers and extremes, respectively.

No distinct difference in *FGFR1* expression across BI-RADS categories was observed (**Figure 4**, **Supplementary Table 1**). However, a positive trend was found between *FGF1* expression and MBD (*P*_trend_=0.06), in which *FGF1* expression was higher among patients in BI-RADS categories B, C and D, compared with BI-RADS A (*P*<0.05, **Figure 4**, **Supplementary Table 1**). Similarly, *FGF11* expression was higher among patients in the BI-RADS D category relative to patients in BI-RADS A, and *FGF18* expression was higher in BI-RADS B, C, and D compared with BI-RADS A (*P*<0.05). The gene expression distribution of all assessable *FGF* ligands in relation to MBD is shown in **Supplementary Table 1**.

## 4 Discussion

Despite being an established independent breast cancer risk factor, knowledge on molecular biology underlying the MBD–breast cancer link is limited. To the best of our knowledge, this is the first study to assess the expression of FGFR1 in paired tumor-adjacent and tumor tissues, and its associations with MBD and tumor characteristics. Herein, we report that paired tumor-adjacent and tumor tissues express FGFR1 differentially, with elevated tumor-specific FGFR1 levels present in nearly 60% of the patients. High FGFR1 expression in tumor tissues is associated with less favorable tumor characteristics, and *FGF* ligand (*FGF1, FGF11, FGF1*8) expression is associated with MBD.

Discovery of MBD-related mediators may have implications on breast cancer prevention as MBD has recently been shown as a modifiable risk factor for breast cancer. *FGFR1* amplification is described as an early event in breast cancer initiation and dysregulated FGFR1 signaling is frequently occurring in breast cancer, and FGFR1 is a target for investigation in ongoing clinical trials for breast cancer therapy (17). Due to its role in paracrine signaling between epithelial cells and stromal fibroblasts that is emphasized in an ECM-dense environment, FGFR1 has been proposed as an MBD-related mediator in breast cancer development (12, 15).

Previously, a higher rate of *FGFR1* amplification was reported in invasive breast carcinoma relative to ductal carcinoma *in situ*, particularly in high grade tumors (27) with *FGFR1* amplification present in up to 14% of breast cancer patients (16, 28). However, the increase in copy number may not translate into a proportional increase in protein expression levels (29). In the present study, we found tumor-specific FGFR1 levels to be increased in 58% of the patients with early breast cancer compared with the corresponding FGFR1 levels in paired tumor-adjacent tissues.

Our findings further show an association between high tumor-specific FGFR1 levels with histological grade 3, high Ki67 index and Luminal B-like tumors. Consistently with our findings, *FGFR1* amplification was previously shown to be associated with high Ki67 (30). Higher frequencies of *FGFR1* amplification were also observed in luminal B-like tumors (31, 32), ER+, HER2-negative breast cancers as well as in patients >50 years of age (33). *FGFR1* amplification was also reported to be associated with resistance to endocrine therapy, specifically, tamoxifen (34). FGFR1 activation in the mammary epithelium was shown to stimulate proliferation and upregulate epithelial-to-mesenchymal transition (EMT) in a HER2+ breast cancer model (35). An *in vitro* study that employed a luminal B cell line (BT-474) also showed a dependence on FGFR1 activation for cell proliferation through PI3K/AkT (36).

The rationale behind staining tumor-adjacent tissues was to assess the association between MBD measurements and the changes in the breast tissue expression profile between tumor-adjacent and tumor tissue. However, in our cohort, no association between FGFR1 expression in tumor-adjacent or tumor tissues and MBD was established. FGF ligands are secreted by activated fibroblasts, including mammary fibroblasts, and stored in the local microenvironment bound to extracellular matrix where they can induce FGFR1 signaling in adjacent epithelial cells (37). In our study, *FGF1* expression in breast tumors was positively associated with MBD. In contrast to the present results addressing *FGF* ligand expression within the breast tumor, a study assessing circulating growth factor levels showed no association between plasma *FGF1* expression and MBD (38). *FGF11* and *FGF18* were also found to be associated with MBD in our cohort, however limited information exists on their role in MBD or breast cancer. FGF11 is classically known as an intracellular FGF, although a recent study described extracellular activity of FGF11 and the capacity to induce cell proliferation *via* activation of FGFR1 (39). *FGF18* copy number and mRNA levels have previously been shown to be increased in breast cancer compared with normal breast tissue (40). An *in vitro* study showed that FGF18 was induced during hypoxia and involved in cell cycle regulation and migration of breast cancer cells (40). A recent study showed that high levels of another FGFR1 ligand, FGF19, in breast microdialysis samples are strongly correlated with high MBD (41). However, this result could not be confirmed at the gene expression level in the present cohort with only insignificantly higher levels noticed among patients with the highest MBD.

A major strength of our study is the use of a well-characterized large prospective study population with validated clinical information. Another unique strength is the availability of paired tumor-adjacent and tumor tissues which provided us the opportunity to analyze differences in FGFR1 levels in-between breast epithelial and cancer cells. However, no information was available on the distance from the tumor adjacent tissue to the tumor border. Access to both FGFR1 gene and protein expression data is another strength as well as access to *FGF* ligand expression data.

The study also has some limitations. While TMA allows for protein expression analyses in a large number of samples, it does not represent the entire tumor tissue. However, the use of duplicate tissue cores reduces the influence of potential tissue heterogeneity. Despite the high number of participants of the KARMA cohort, the short follow-up time limited the number of incident breast cancer patients to study, and the final subgroup analyses were still restricted by small sample sizes that could have affected the robustness of the tests. The study is based on a large prospective mammography screening cohort and the present study population comprises breast cancer patients in Sweden, where the median age at diagnosis is 64 years on a national level, with few premenopausal patients. Therefore, our results are mainly restricted to postmenopausal women. As breast density varies according to age, the study results are not generalizable to younger women.

In conclusion, the present study showed increased FGFR1 expression in breast tumor tissue compared with paired tumor-adjacent breast tissue from the same patient. No clear association between FGFR1 expression and MBD was established. FGFR1 expression in tumor tissues was associated with less favorable tumor characteristics. *FGF-1, -11, -18* expression in tumor tissues was associated with MBD. Further studies are needed to better understand the contribution of the FGFR1/FGF system to the molecular mechanisms behind the influence of MBD on breast cancer risk.

## Data availability statement

The data analyzed in this study is subject to the following licenses/restrictions: The datasets presented in this article are not readily available because of legal, ethical and privacy restrictions to protect patient confidentiality. Requests to access these datasets should be directed to the corresponding author (AR) and the steering committees of the KARMA and SCAN-B studies (http://www.karmastudy.org/ and https://www.scan-b.lu.se/).

## Ethics statement

The studies involving human participants were reviewed and approved by the regional Ethics Committee at Karolinska Institutet. The patients/participants provided their written informed consent to participate in this study.

## Author contributions

Conceptualization, AR; formal analysis, ÖB and AR; resources, MK, JV-C, PH, SB, and AR, writing–original draft preparation, ÖB; writing–review and editing, ÖB, MK, JV-C, SZ, PH, SB, and AR; supervision and funding acquisition, PH, SB, and AR. All authors contributed to the article and approved the submitted version.
