# Supplementary material for: FGF/FGFR1 system in paired breast tumor-adjacent and tumor tissues, associations with mammographic breast density and tumor characteristics
Source: Front Oncol. 2023 Jul 20;13:1230821. doi: 10.3389/fonc.2023.1230821 (PMC10400328; doi:10.3389/fonc.2023.1230821)
Supplement: Supplementary file 1 [file Table_1.docx]

Supplementary Material

FGF/FGFR1 system in paired breast tumor-adjacent and tumor tissues, associations with mammographic breast density and tumor characteristics

**Öykü Boraka^1^, Marie Klintman^1^, Johan Vallon-Christersson^2^, Sophia Zackrisson^3^, Per Hall^4,5^, Signe Borgquist^1,6^, Ann H Rosendahl^1*^**

*** Correspondence:** Ann Rosendahl ann.rosendahl@med.lu.se

**Supplementary Table 1.** Distribution of *FGFR1* and *FGF* ligand expression across BI-RADS MBD categories.

|  | **BI-RADS category for mammographic breast density** | | | | ***P*_trend_ *** |
| --- | --- | --- | --- | --- | --- |
|  | **A** | **B** | **C** | **D** |  |
| *FGFR1* | -0.20 (-0.80–0.85) | 0.02 (-0.39–0.46) | 0.03 (-0.33–0.39) | 0.09 (-0.32–0.40) | 0.52 |
| *FGF1* | -0.39 (-1.49–0.28) | **0.08 (-0.52–0.69)^a^** | **0.21 (-0.46–0.67)^a, b^** | **0.32 (-0.45–1.07)^a, b^** | 0.06 |
| *FGF2* | 0.13 (-1.07–1.27) | 0.03 (-1.05–0.98) | -0.07 (-0.91–0.76) | -0.26 (-0.95–1.00) | 0.56 |
| *FGF5* | -0.13 (-0.18–0.24) | -0.18 (-0.18–0.10) | -0.18 (-0.18–0.01) | -0.18 (-0.18–0.21) | 0.93 |
| *FGF7* | 0.21 (-1.43–0.64) | 0.18 (-0.58–0.83) | 0.06 (-0.60–0.52) | 0.10 (-0.50–0.72) | 0.36 |
| *FGF9* | -0.29 (-0.63–0.13) | -0.20 (-0.67–0.40) | -0.19 (-0.67–0.41) | -0.38 (-0.67–0.56) | 0.88 |
| *FGF10* | -0.40 (-1.25–0.62) | -0.18 (-1.21–0.95) | -0.19 (-1.37–0.68) | 0.12 (-0.94–1.27) | 0.73 |
| *FGF11* | -0.34 (-1.33–0.54) | -0.08 (-0.75–0.68) | 0.04 (-0.71–0.60) | **0.28 (-0.72–0.80)^a^** | 0.24 |
| *FGF16* | -0.79 (-1.71–0.31) | **0.27 (-0.93–1.05)^a^** | -0.29 (-1.25–1.00) | -0.05 (-1.06–0.54) | 0.45 |
| *FGF18* | -0.84 (-1.32–0.47) | **0.03 (-0.57–0.87)^a, b^** | **0.07 (-0.74–0.85)^a^** | **0.40 (-0.86–0.92)^a^** | 0.62 |
| *FGF19* | -0.29 (-0.29–0.02) | -0.29 (-0.29–0.10) | -0.29 (-0.29–0.22) | -0.06 (-0.29–0.48) | 0.09 |
| *FGF20* | -0.32 (-0.32–0.02) | -0.32 (-0.32–0.34) | -0.32 (-0.32–0.32) | -0.32 (-0.32–0.43) | 0.18 |
| *FGF22* | -0.37 (-0.37–(-0.15)) | -0.37 (-0.37–0.31) | **-0.28 (-0.37–0.29)^a^** | -0.37 (-0.37–0.01) | 0.62 |

Gene expression values shown as median (IQR).

*Joncksheere-Terpstra test.

^a^Pairwise comparison with reference to category A (*p*<0.05).

^b^Pairwise comparison with reference to category A, with Bonferroni correction (*p*<0.05).
